# Co-delivery of brinzolamide and miRNA-124 by biodegradable nanoparticles as a strategy for glaucoma therapy

**DOI:** 10.1080/10717544.2020.1731861

**Published:** 2020-03-05

**Authors:** Tingting Li, Ye Wang, Jiahao Chen, Xiaoshu Gao, Siqi Pan, Yu Su, Xinrong Zhou

**Affiliations:** a Shanghai General Hospital, National Clinical Research Center for Eye Diseases, Shanghai Key Laboratory of Ocular Fundus Diseases, Shanghai Engineering Center for Visual Science and Photomedicine, Shanghai engineering center for precise diagnosis and treatment of eye diseases, Shanghai, China;; b Department of Ophthalmology, Peking University Shenzhen Hospital, Shenzhen, China;; c Department of Biological pharmacy, School of Pharmacy, Jilin University, Changchun, China

**Keywords:** Glaucoma, miRNA-124, nanoparticle, brinzolamide, IOP, pharmacodynamics, neuroprotection

## Abstract

Co-delivery nanoparticles with characteristics of intracellular precision release drug have been generally accepted as an effective therapeutic strategy for eye diseases. In this study, we designed a new co-delivery system (miRNA/NP-BRZ) as a lasting therapeutic approach to prevent the neuro-destructive after the long-term treatment of glaucoma. Neuroprotective and intraocular pressure (IOP) response were assessed in *in vivo* and *in vitro* models of glaucoma. At the meaning time, we describe the preparation of miRNA/NP-BRZ, drug release characteristics, intraocular tracing, pharmacokinetic and pharmacodynamics study and toxicity test. We found that miRNA/NP-BRZ could remarkably decrease IOP and significantly prevent retinal ganglion cell (RGC) damages. The new formula of miRNA-124 encapsulated in PEG-PSA-BRZ nanoparticles exhibits high encapsulation efficiency (EE), drug-loading capacity (DC), and stable controlled-release efficacy (EC). Moreover, we also verified that the miRNA/NP-BRZ system is significantly neuroprotective and nontoxic as well as lowering IOP. This study shows our co-delivery drug system would have a wide potential on social and economic benefits for glaucoma.

## Introduction

Glaucoma refers to a group of neurodegenerative eye disorders that cause vision loss and irreversible blindness globally (Weinreb et al., 2014). It is estimated that the number of people with glaucoma will go up to 80 million in 2020, and about 111.8 million people will be affected worldwide by 2040 (Quigley & Broman, 2006). Glaucoma is classified into chronic primary angle-closure glaucoma (PACG) and primary open-angle glaucoma (POAG; Weinreb et al., 2014). Although more than 80% of cases are POAG, PACG is responsible for the vast majority of glaucoma blindness in China (Foster & Johnson, 2001; Quigley & Broman, 2006). Glaucoma is mainly associated with increased intraocular pressure (IOP; Weinreb et al., 2014). Pupillary block is the most common mode of angle closure and is caused by blockage of aqueous humor outflow from posterior to anterior chamber at the pupil, which in turn leads to very high IOP resulting in optic nerve damage at a later stage (Lachke et al., 2011; Weinreb et al., 2014). In PACG, reduction of IOP through conventional medical and surgical intervention is the only proven method. However, the lack of neuroprotective treatments causes apoptosis of retinal ganglion cells (RGCs). Glaucoma is an optic neuropathy characterized by progressive degeneration of RGCs apoptosis, eventually resulting in optic disc damage and visual loss (Welsbie et al., 2013). Due to the temporariness and repeatability of operation, drug therapy still accounts for most of the glaucoma treatments. Brinzolamide that is a carbonic anhydrase inhibitor could be a common clinical drug to lower the IOP, and its effect of reducing IOP is still shown in recent researches (Moosavi & Ansari, 2018; Liu et al., 2019). Sufficient preclinical and clinical studies support that miRNA has become potential therapeutic agents for various diseases so far (Cao et al., 2016). It has been shown that many identified miRNAs display spatio-temporal patterns of expression during early human brain development (Cao et al., 2016). Furthermore, lots of studies have demonstrated that specific miRNAs have versatile functions in both invertebrates and vertebrates during neural development and brain activities (Davis et al., 2015). Accordingly, miR-124 is the most abundant miRNA in the CNS and can be transcribed from three different loci, including miRNA-124-1, miRNA-124-2, and miRNA-124-3 (Lagos-Quintana et al., 2002; Sun et al., 2015). Neuronal differentiation and anti-inflammatory effects of early miR-124 treatment were recently shown to modulate the polarization of activated microglia and infiltrating macrophages toward the anti-inflammatory M2 phenotype and protect neurons in various ways after nervous system diseases (Hamzei Taj et al., 2016). Therefore, reducing the susceptibility of RGCs to apoptotic stimuli may have the potential to strengthen medical effects by neuroprotective agents.

Nano-sized drug carriers have been conducted in the field of ophthalmology (Kim et al., 2009; Lee et al., 2017; Liao et al., 2017; Rathor et al., 2017). There are at least five prevalent categories of nanoscale delivery systems including viral capsids, lipid-based nanoparticles, peptide-based nanoparticles, polymer-based nanoparticles, and silicon nanoparticles. The eye, especially the posterior segment, is composed of tissues that are difficult for drugs to penetrate due to structural peculiarities arising from its unique anatomy and physiology. Carrier materials such as poly SA (PSA) and polyethylene glycol (PEG), polyanhydrides which are biodegradable, capable of controlled-release, and nontoxic are generally selected (Brem et al., 1989; Laurencin et al., 1990). The polymer-based nanoparticle systems show promise as active vectors that can deliver the drug to retina via intraocular administration. Moreover, the drugs encapsulated in nanoparticles are capable of penetrating deeply into the targeting regions while enabling the safe and efficacious medication compared to the standard formulations (Suk & Gopinath, 2017). In addition, the encapsulated drug delivery systems enable the nanoparticles to have suitable particle size, surface properties and ability to cross the vitreous body smoothly, ensuring the effectiveness of drugs (Rathor et al., 2017; Suk & Gopinath, 2017). High polymer graft copolymerization technology was used for polymerization of two polyanhydrides (Rathor et al., 2017). Based on the above points, we designed a nanoparticle (NP) drug-loading system where drug molecules are encapsulated with the aid of carrier materials that can help overcome issues such as poor bioavailability, high drug dosage, and short drug half-life. A drug delivery system involving nanoparticles and miRNA has improved bio-distribution and lower toxicity which can mitigate the problems faced during ocular delivery (Suk & Gopinath, 2017). Polymer-based nanoparticles are undoubtedly privileged for eye diseases. Herein, the combinational treatment of brinzolamide and mi-RNA-124 delivered via the nanoparticle pattern for the preventing optic nerve damage and lowering IOP is a potent strategy to resolve the adverse effects of previous treatments.

Our approach is focused on the development of nanoparticles for targeted ophthalmic miRNA delivery based on our previous studies (Lei et al., 2016; Li et al., 2018). We demonstrated PEG-PSA nanoparticles carrying BRZ and miRNA-124 were delivered into the retina by intravitreal injections and this procedure successfully accomplished adhesive retention in the trabecular outflow pathway to introduce BRZ and miRNA-124 into the retina with significant efficacy and neuroprotection. Thus, the present study reveals that may serve as a novel potent therapeutic strategy for optic neuroprotection and functional recovery after onset of glaucoma.

## Materials and methods

### Materials and instruments

#### Reagents

Brinzolamide (BRZ) (China Chen Xi Pharmaceutical Institute, batch number: 201503); PEG (medical grade, Hebei Huanghua Xinnuolixing chemical stock corporation, average relative molecular weight: 2000** **Da); SA (Sigma-Aldrich, St. Louis, MO); 1% tropicamide eye drops (Santen, Japan); anesthetic drop TobraDex (Alcon, USA); rabbit polyclonal Tuj-1 antibody (1:100, Proteintech, Rosemont, IL); 5% chloral hydrate, 4% triformol, penbritin, and DAPI (40,6-diamidino-2-phenylindole) (Sigma-Aldrich, St. Louis, MO); hematoxylin solution (M type) and 0.3% eosin alcoholic solution (Muto Pure Chemicals, Tokyo, Japan); mounting medium (Vectashield, Vector Laboratories Inc., Burlingame, CA). The miRNA expression plasmids for miRNA 124, pcDNA 3.2/V5-hsa-rni-124a were purchased from Addgene (Addgene plasmid 26306), and other chemical reagents were analytically pure.

#### Experimental animals

A total of 30 dutch-belted rabbits and 69 C57/BL6 mice were provided by the Experiment Animal Center, Jilin University.

#### Instruments

Thirty-gauge and 33 gauge injection needles (Hamilton, Reno, NV); BK30 Hereus sepatech (German Sigma Corporation); Zeta nanosizer (Zetasizer Nano ZS, British Malvern Instruments); transmission electron microscope (HitachiS-4800N, Hitachi, Japan); HP1100 HPLC (Hewlett Packard, Palo Alto, CA); pathological section machine (Leica, Bannockbum, IL); fluorescence microscope (DMI4000B; Leica, Germany); ophthalmotonometer (Reichert, Depew, NY 14043); image analysis system (Imagepro Plus 6.0; Cybernetics, Bethesda, MD).

### Synthesis of PEG-PSA-BRZ

**Figure 1.**
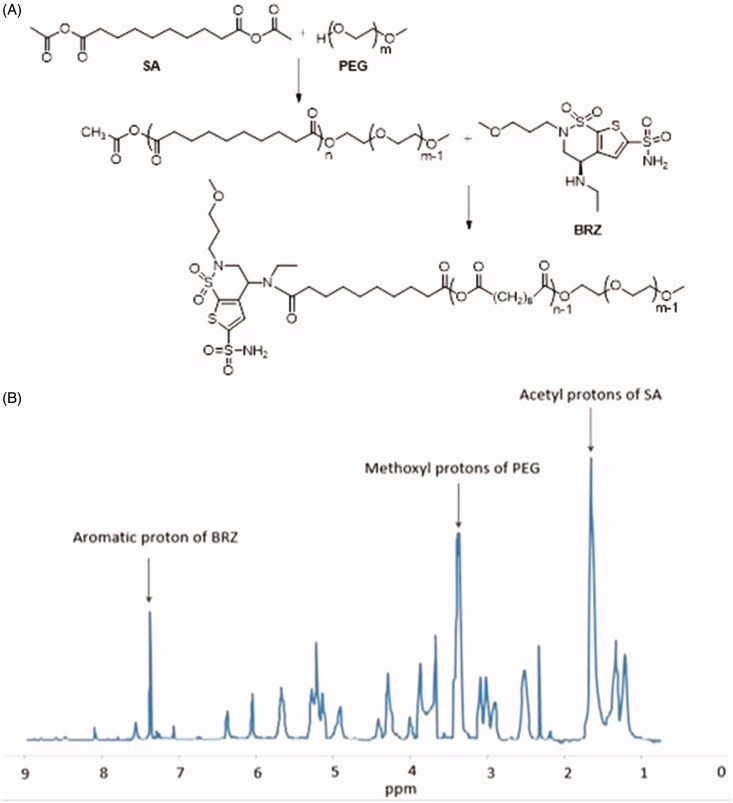
(A) The flow of synthesizing carrier materials, the amphiphilic polymers PEG-PSA-BRZ; (B) ^1^H NMR spectrum of PEG-PSA-BRZ.

Sebacic anhydride (Acyl-SA) was synthesized as reported previously (Fu et al., 2002). PEG methyl ether was lyophilized to constant weight. Predefined ratios of Acyl-SA and PEG were melted (105** **°C) in the oil bath pot for 2** **h before applying high vacuum for at least 30** **min. The diblock poly ethylene glycol-co-polysebacic acid (PSA-PEG) were synthesized by melt polycondensation of Acyl-SA and PEG. The products were washed in ether and kept for a certain time period for organic solvent to be volatilized. Then, the products were lyophilized and dissolved in a mixture of dichloromethane, DMSO, and acetone. To form PEG-PSA-BRZ, BRZ and carboxyl of PSA were condensed together. EDC and BRZ (whose activity had been enhanced by acyl chloride) were added in equal molar ratio into the mixture and then sealed for 24** **h. After the reaction was finished, the product was washed with ice cold ether, and lyophilized completely after the organic solvent had been removed. The reaction was shown in Figure 1(A). Polymer molecular weight was determined by gel permeation chromatography (GPC) and polymer structure was characterized by using ^1^H NMR (Figure 1(B)).

### Preparation of nanoparticles

According to previous studies (Zhang et al., 2011; Han et al., 2015), nanoparticles were prepared by emulsification-solvent evaporation method. 200** **mg of the dry carrier materials, PEG-PSA-BRZ, were dissolved in a 5** **mL mixture of DMSO and methylene chloride and subjected to an ultrasonic device after half an hour of incubation. 100** **µg of miRNA-124 plasmid was dissolved in a mixture of water and DMSO and then subjected to an ice bath under 450** **W ultrasonic waves for 4** **min. Thereafter, the organic solvent was removed entirely under vacuum. The residue was centrifuged at 2000** **×** ***g* for 15** **min to collect the nanoparticles. After lyophilization, the physicochemical properties of the nanoparticle system (i.e. miRNA/NP-BRZ) were measured before biological evaluation.

### Measurement of physico-chemical properties

An appropriate amount of freeze-dried nanoparticle powder (miRNA/NP-BRZ) was taken and observed under transmission electron microscope (TEM). The nanoparticle emulsion was diluted 10 times with distilled water at room temperature and the particle size distribution of nanoparticles was measured by the Zetasizer Nano ZS analyzer (Wu et al., 2016).

### Encapsulation ratio and drug loading capacity

UV spectrophotometric method was used to determine the encapsulation ratio and drug-loading capacity of nanoparticles. BRZ was tested under 254** **nm. The concentration of BRZ was determined by HPLC method after extraction and purification of nanoparticles (Pradhan et al., 2015; Tang et al., 2015). The chromatographic conditions for detection of BRZ were as follows (Hassib et al., 2016); mobile phase: ethanol:methanol:n-hexane (55:5:40); flow rate: 1.0** **mL/min; column temperature: 25** **°C; detection wavelength: 254** **nm; injection volume: 20** **µL; and retention time: 6.2** **min.

The nanoparticles were weighed accurately and dissolved it in dichloromethane. They were extracted using water, thrice, and then partitioned in a mixture of methanol and n-hexane, thrice, in order to obtain the fat-soluble BRZ. 20** **µL was taken and the peak area was determined. The standard curve was plotted to calculate its concentration.

Formulas for calculating encapsulation efficiency (EE) and drug-loading capacity (DC) were as follows:

(1) EE %=[(Drug added–Free “unentrapped drug”)/Drug added]×100

(2) DC %=(Actual drug loading/weight of Nanoparticles)×100

(3) Encapsulation efficiency %=[(Drug added–Free “unentrapped drug”)/Drug added]×100

### Drug release and kinetic study in vitro

The nanoparticle solution was dispersed at a concentration of 2** **mg/mL and was incubated in phosphate buffer solution (PBS) (pH = 7.4). The solution was stirred at 37** **°C and after a specific interval time, it was centrifuged and the supernatant was collected. HPLC was done to measure the concentration of BRZ and miRNA-124.

The chromatographic conditions for detection of miRNA-124 were as follows: the column used was Agilent C18 (4.6** **mm × 250** **mm, 5** **µm); mobile phase: methanol:water (5:95), flow rate: 1.0** **mL/min, column temperature: 25** **°C, injection volume: 20** **µL, and retention time: 19.5** **min.

The chromatographic conditions for detection for BRZ were as follows: the column used was Agilent C18 (4.6** **mm × 250** **mm, 5** **µm), mobile phase: ethanol:methanol:n-hexane (55:5:40), flow rate: 1.0** **mL/min, column temperature: 25** **°C, detection wavelength: 254** **nm, injection volume: 20** **µL, and retention time: 6.2** **min.

In the present study, we used Dutch-belted rabbits. The rabbits were anesthetized by intramuscular injections of a mixture of 25** **mg/kg ketamine and 2.5** **mg/kg xylazine and 1% tropicamide was applied for mydriasis. 100** **µL of PEG-PSA-BRZ nanoparticles (containing 1.15** **mg BRZ and 10** **µg miRNA-124, respectively) were injected intraocularly into one eye after dripping 0.5% proparacaine hydrochloride into it. The same amount of saline was injected into the other eye. 100** **µL of aqueous humor was collected for examination after 1, 3, 5, 7, 9, 11, 13, 15** **days by inserting a 30-gauge needle into the anterior chamber of anesthetized rabbit. On the 15th day, the vitreous samples were taken and stored at −80** **°C until further use.

### Intraocular tracing of miRNA/NP-BRZ

C57/Bl6 mice, which were anesthetized by 5% chloral hydrate, were intravitreally injected with miRNA/NP-BRZ which was encapsulated along with a fluorescent tag, DIR (Mohd Salleh et al., 2014). Specific operations were performed as follows: 1) the conjunctiva was exposed using ophthalmic forceps; 2) Approximately, 3** **µl of prepared miRNA/NP-BRZ with fluorescent tags was injected intravitreally by a 33 gauge needle into the back of ciliary body; 3) the eyeballs were removed at 6, 24, or 48** **h after the operation, and fixed with 4% paraformaldehyde at 4** **°C overnight; 4) the eyeballs were soaked in 30% sucrose for 14** **h at 4** **°C to cryoprotect them; 5) the eyeballs were then embedded into optimal cutting temperature (OCT) embedding medium (Sakura Finetek, Torrance, CA), frozen in liquid nitrogen and used for preparation of 6-µm-thick sections by cutting through optic nerve head; and 7) the images were acquired using a fluorescent microscope (DMI400B; Leica, Germany) with a 400× objective lens.

### In vitro pharmacodynamics study

In this study, we performed a procedure where small volumes of polystyrene microspheres were injected into the anterior chamber to impede aqueous outflow and elevate IOP and 69 mice were used for this procedure. The baseline IOP of each eye was measured a day before the operation. Firstly, the animals were anesthetized with 5% chloral hydrate, the pupils were enlarged with 1% tropicamide eye drops (Santen, Japan) and TobraDex (Alcon, USA) anesthetic drops were added to each eye. A 33-gauge needle was inserted into the anterior chamber through the corneal center and 2** **µm polystyrene of the microsphere’s solution was injected. In order to avoid contact between the needle and lens, the needle was inserted at an angle of 45° making sure that the vitreous was filled appropriately. Then, 2** **µL of air was injected before pulling the needle to seal the small puncture. On day 3, 2** **µL miRNA/NPs-BRZ and 2** **µL PBS was injected intravitreally. The control group was only punctured in vitreous region without injection. The baseline IOP was recorded before the injection of polystyrene microspheres at the same time each day using the ophthalmotonometer (Reichert, Depew, NY 14043). The normal IOP of each eye was expressed as an average of values measured by 10 ophthalmotonometers (Suman et al., 2014).

### Optic nerve protection efficacy in vivo

We used mice models to study optic nerve injury. All the animals used in this study had optic nerve injuries in their left eyes. The mice were divided into three groups: optic nerve crush (ONC) and injection with nanoparticles and miRNA/NP-BRZ intravitreally (69 mice, ONC + miRNA/NP-BRZ group); ONC and injection with PBS intravitreally (69 mice, ONC + PBS group); and ONC and vitreous punctured without injection (27 mice, ONC only or negative control). The mice were anesthetized by intraperitoneal injection of 5% chloral hydrate. With the help of an operating microscope, a lateral canthotomy was performed in the left eye of the mice. The conjunctiva was incised laterally to the cornea, the muscle was separated, and the optic nerve was exposed. Care was taken to avoid damaging the small vessels around the optic nerve. Force-controlled forceps were applied to the optic nerve 1** **mm posterior to the globe for 10** **sec in each animal (Li et al., 1999). The eyelids were shut after the surgery. In all cases, we verified that the integrity of the retinal blood supply was not damaged. After ONC, miRNA/NP-BRZ group mice were injected miRNA/NP-BRZ dissolved in PBS into the vitreous. A 30-gauge needle was inserted into vitreous through slightly into the back of ciliary body and 3** **µL of miRNA/NP-BRZ solution was injected. PBS group eyes were treated the same way using 3** **µL of PBS. The control group mice only received vitreous puncture. Ampicillin was applied to the wound to prevent bacterial infection.

### Fluorescent immunohistochemical staining of Tuj-1

The mice were sacrificed using a systemic injection of 4% paraformaldehyde 3** **days after the ONC. The eyes were removed and fixed in PBS (pH = 7.4) containing 4% paraformaldehyde for 24** **h. Then, the eyes were embedded onto Tissue-Tek^®^ and slices of 6** **µm were made by sectioning through the cross-section of the optic nerve head. The slices were washed three times in PBS for 15** **min each time and were incubated at room temperature for 1** **h in 0.1% Triton-X100 PBS containing 10% donkey serum. The slides were incubated at 4** **°C overnight with rabbit polyclonal Tuj-1 antibody (1:100, Proteintech, Rosemont, IL) to stain the GCL. After washing with PBS three times at room temperature, the slides were incubated for 60** **min with the secondary antibody donkey-anti-rabbit TRITC (1:1000; Abcam, Eugene, OR) (Ko et al., 2016). The slices were washed again with PBS and nuclear staining with DAPI was done to facilitate the orientation on the slides. Fluorescence microscope (DMI4000B; Leica, Germany) was used for taking images of retina and image analysis system (IMAGEPRO PLUS 6.0; Cybernetics, Bethesda, MD) was used for analysis. All the cells in GCLs were checked in each mouse. The number of Tuj-1 positive cells was counted continuously one by one under the high-power fields (HPF, 400 magnification).

### Measurement of RGC density

For the detection of survival RGCs in GCL, RGCs were retrogradely labeled with fluorogold. Fluorogold was injected into the superior colliculi on day 4 before the ONC and vitreous injection. This technique was chosen to avoid confounding the RGCs with the dye engulfing the microglia or macrophages. As described in detail previously (Ryu et al., 2012), 2** **μL of 4% fluorogold (Abcam, Eugene, OR) was injected into superior colliculi of each hemisphere after a craniotomy was performed. After the tracer injections, the skin was re-sutured. The procedures for the ONC and vitreous injection were described above. On day 3 after the ONC and vitreous injection, the mice were sacrificed, and their complete retina were placed on glass slides with the ganglion cell layers facing upward. RGC density was determined by counting tracer-labeled RGCs in 12 distinct areas under a microscope as previously described (Himori et al., 2013). The number of RGCs was counted in a masked manner using the IMAGEPRO PLUS 6.0 software (Cybernetics).

### TUNEL staining

Three days after the injection, TUNEL staining was performed on the cross-section of retina to record the number of TUNEL positive cells (i.e. Tuj-1^+^ cells) in GCL. Later, the apoptotic cells were determined using TUNEL staining with in-situ cell death detection kit (Roche Diagnostics Corp, Indianapolis, IN). As described before, the slices were washed three times in PBS for 15** **min and stained following the manufacturer’s protocol. The TUNEL positive cells in GCL of each sample were counted by HPF (400 magnification) to assess the apoptosis index. Each group had 5 mice.

### Real-time PCR analysis

Three days after intravitreal injection, the total mRNA was extracted from retina homogenates by using the Trizol (Qiagen, Valencia, CA) according to the manufacturer’s instructions. The RNA concentration was determined using a NanoDrop ND-2000 spectrophotometer (Thermo, Waltham, MA). Purified mRNA (1** **µg) was used for the first-strand cDNA synthesis using FastQuant RT Kits (KR106, Tiangen, Beijing). The real-time PCR program was as follows: 94** **°C for 10** **s, followed by 40 cycles of 94** **°C for 5** **s, 60** **°C for 30** **s for annealing, and 72** **°C for 15** **s. Each cDNA template was amplified in triplicate using SYBR Green PCR Master Mix (FP205, Tiangen, Beijing). The PCR reaction was directly monitored using a genetic analyzer (ABI PRISM 7300 System; Applied Biosystems, Waltham, MA). The primers were the following: Thy1.1, F: G G T G G C A G A A G A A G A C A A G G A G, R: G G G C A A G G G A A A G A A G A A T A A A G G; Nefh, F: C C C T C C G T A A G A A G A A A C A C T G, R: C G T A G C G T T C A G C A T A C A T C A C. The expression level of genes was calculated by using the 2^−ΔΔCt^ analysis method.

### Statistical method

Paired *t*-test was used to compare the parameters between the drug group and control group. The relationship between rate of RGC loss in the drug group and IOP exposure was analyzed by SPSS16.0. The difference was considered statistically significant when *p*** **<** **.05.

### Evaluation of miRNA/NP-BRZ toxicity

To evaluate the toxicity of nanoparticles, we examined the retina tissue slice with nanoparticles. Hematoxylin and eosin (H&E) staining of the cryosections from the posterior segment of the eye was performed on day 1, 2, and 3. Following the sacrifice, the eyeballs were removed and processed for histopathology, including fixation in 4% paraformaldehyde, embedding in OCT compound and H&E staining using the standard techniques. For H&E staining, the slice thickness was set at 6** **µm and the optic nerve head area could be found in sections when examined under a microscope (Carl Zeiss). The retina from each animal was imaged and Photoshop software was used to perform quantitative analysis of the cell numbers. The density of cells in GCL was counted and calculated in 10 high-power fields (HPF 400 magnification).

## Results

### Physicochemical properties of miRNA/NP-BRZ

**Figure 2.**
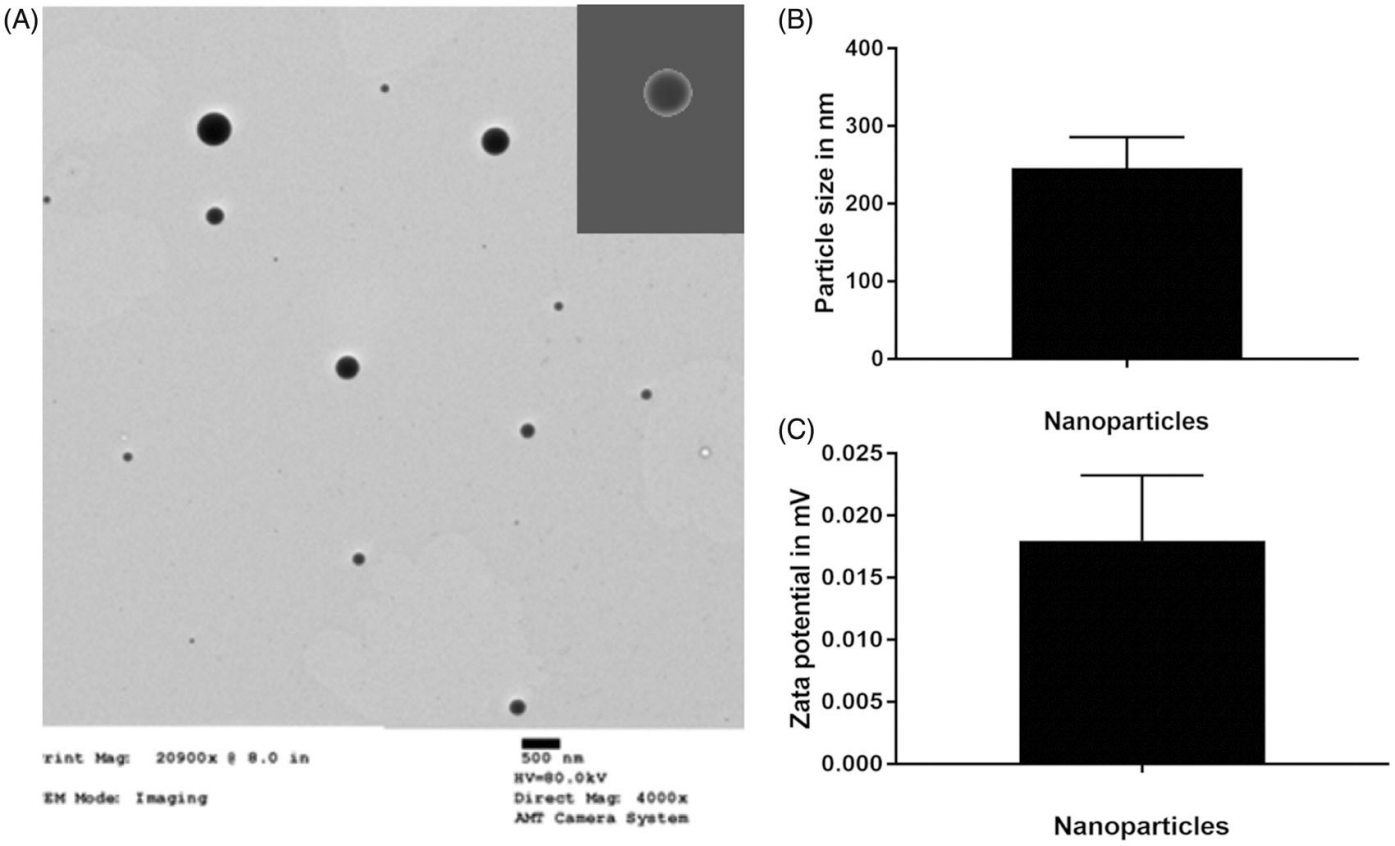
(A) TEM image of PEG-PSA nanoparticles; (B) Statistical particle size of PEG-PSA nanoparticles; (C) Zeta potential (mV) of PEG-PSA nanoparticles.

The PEG-PSA-BRZ nanoparticles prepared by emulsification-solvent volatilization method were observed under a transmission electron microscope. The size of nanoparticles was in the range from 250 to 400** **nm and the appearance of nanoparticles is shown in Figure 2, which was deemed suitable for intraocular injection (Shah et al., 2014). In addition, zeta potential analysis exhibited that the surface charge of nanoparticles was almost neutral, which would greatly reduce the absorption of nonspecific proteins. The encapsulation rate and drug loading efficiency were 90% and 6%, respectively.

### Release characteristics and dynamics in vitro

**Figure 3.**
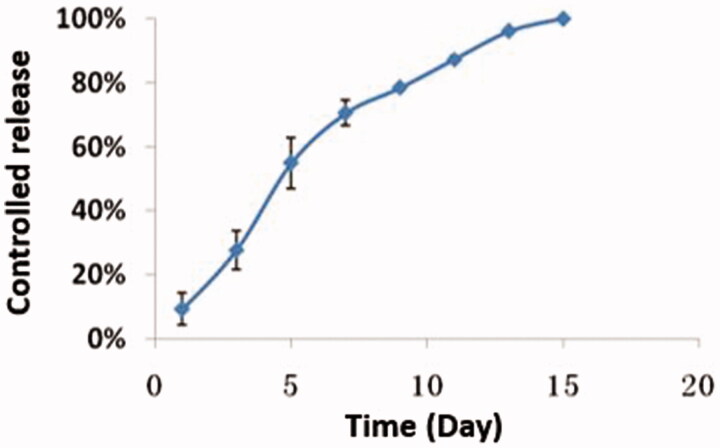
The Cumulative release of nanoparticles.

The release characteristics of the nanoparticles *in vitro* were reflected by the release of the drug. The release curve was drawn accordingly and is shown in Figure 3. As demonstrated in this figure, the drug release was steady and long-lasting from day 1 to day 12. Defining the total area under the curve (AUC) of miRNA/NP-BRZ released over 15** **days (AUC 0–15) as 100%, the proportions of the AUCs of the miRNA/NP-BRZ released during each interval (AUC 0–t) to the total AUC over 15** **days (AUC 0–15) were considered as the percent of drug released by miRNA/NP-BRZ during each interval. The *in vitro* release profile of miRNA/NP-BRZ formulation showed a cumulative mean release rate of over 50% on day 7, over 90% on day 12, and 100% over the period of 15** **days. The mechanism of action is actually based on properties of nanoparticles. The controlled release can last 15** **days due to miRNA-124 encapsulated on PEG-PSA-BRZ. There might be three mechanisms of drug release for the polymeric drug carriers, including the swelling, enzymatic reaction, and dissociation of the drug (Suk & Gopinath, 2017).

The pharmacokinetic release of drugs encapsulated in nanoparticles was examined in the aqueous humor and the pharmacokinetics of the nanoparticles within the retina was simulated, based on that. The concentration of BRZ was 70** **ng/mL. The drug-loading nanoparticles had the effect of lowering IOP and neuroprotection as well as attenuation of optic nerve injury for at least a week in the high IOP and optic nerve injury animal models. The result of dynamics research was consistent with the release pattern *in vitro*. This provided evidence for trace experiment in intraocular bio-distribution. Moreover, it also was demonstrated that drugs encapsulated by the nano-carriers can reach deeper into the targeting regions without being degraded.

### Intraocular bio-distribution

**Figure 4.**
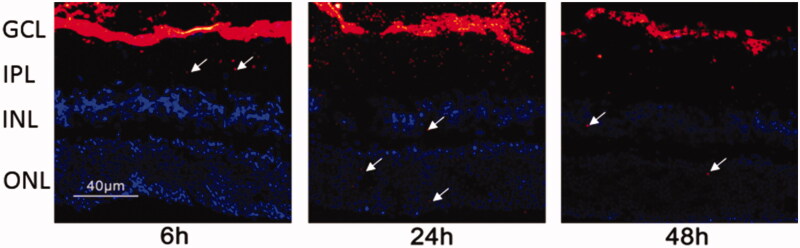
Fluorescent tracing image of the miRNA/NP-BRZ injected into the vitreous body at different time intervals. After 6 h of vitreous injection, the miRNA/NP-BRZ could pass through the vitreous body and reach the fundus of the eye and the GCL layer in the retina. After 24** **h, some of the miRNA/NP-BRZ entered the INL layer and the ONL layer. After 48** **h, nanoparticles could still be found in all layers of the retina. Scale bar: 40** **μm.

According to the instructions for fluorescent tagging using DIR, the distribution of nanoparticles in the retina was evaluated by using confocal microscopy. As depicted in Figure 4, the nanoparticles can cross through the vitreous body and reached the fundus, touching the GCL in the retina at 6** **h after the vitreous injection. At the same time, it can be observed that the minority of nanoparticles went into IPL and INL. At 24** **h after injection, some miRNA/NP-BRZs entered the INL layer and the ONL layer. Moreover, nanoparticles could still be detected in all layers of the retina after 48** **h. The observation demonstrates that miRNA/NP-BRZ is expected to possess good delivery capacity and lasting therapeutic effect. The miRNA/NP-BRZ particles are positively charged (4.3 ± 3.1** **mV) due to the presence of BRZ on the surface, leading to slight decrease in the hydrophilicity.

### Efficiency of the nanoparticles in lowering IOP

**Figure 5.**
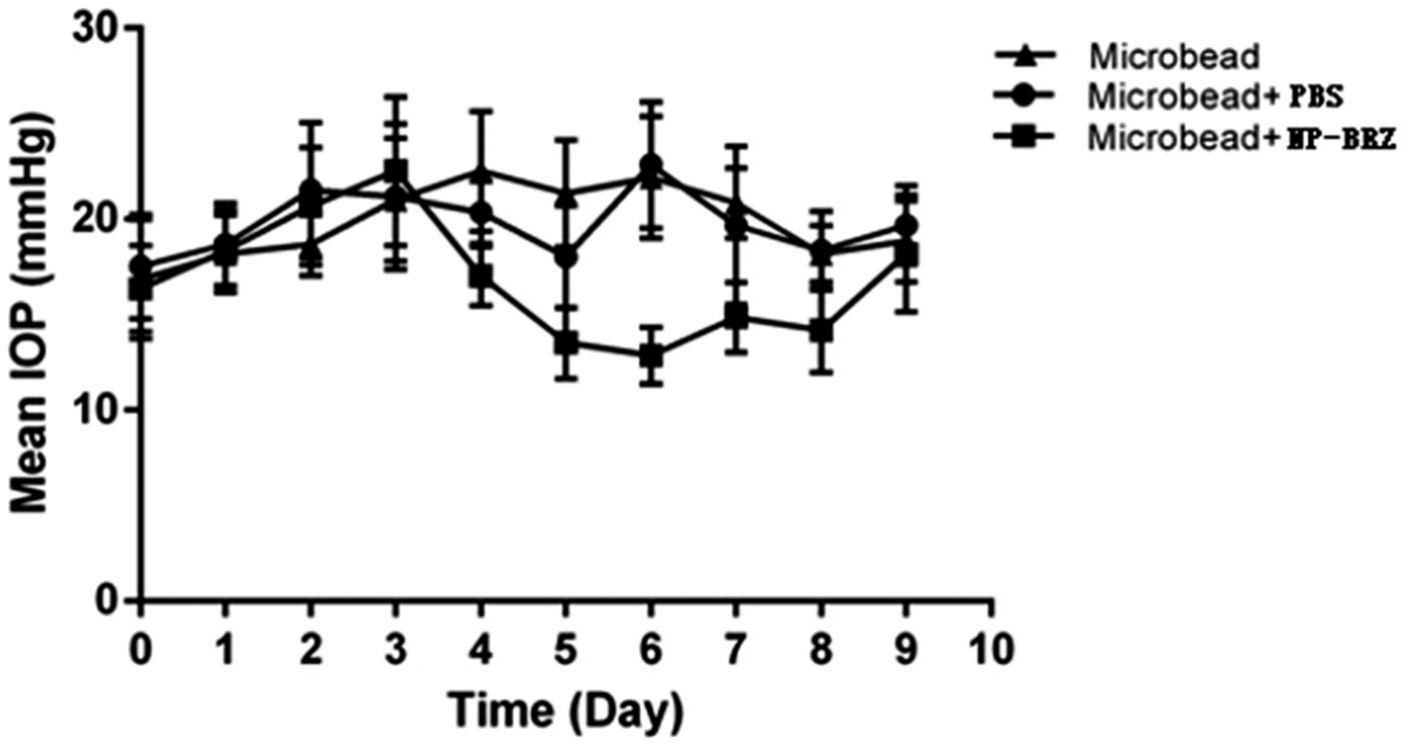
The status of IOP in mice with high IOP after injection of PBS and miRNA/NP-BRZ. There was a significant increase in IOP after the polystyrene microbeads injection (*p*** **<** **.01). On the sixth day, the IOP dropped to minimum in microbead + miRNA/NP-BRZ group. There was a significant reduction in IOP compared to the microbead + PBS group (*p*** **<** **.01) and microbead group (*p*** **<** **.01). Statistical significance was set at *p*** **<** **.05. PBS: phosphate-buffered saline.

Injection of polymer microbeads into the anterior chamber results in the blockage of angle, disturbance of aqueous humor circulation and eventually leading to the elevation of IOP. Thus, a medium height long-term chronic high IOP model was obtained. After injection with the polystyrene microbeads, there was a significant increase in IOP from day 1 to day 3 (Figure 5). On day 3, the IOP increased to 22.50** **±** **3.89** **mmHg (*p*** **<** **.01) in the microbead + miRNA/NP-BRZ group, while IOP in the microbead + PBS group was 21.21** **±** **3.82** **mmHg (*p*** **<** **.01) and microbead group rose to 21.00** **±** **3.22** **mmHg (*p*** **<** **.01). In microbead + miRNA/NP-BRZ group, the IOP began to decrease after the miRNA/NP-BRZ injection. On the sixth day, the IOP dropped to minimum of 12.83** **±** **1.47** **mmHg, which was a significant decrease in IOP compared to the IOP of 2 2.83** **±** **3.31** **mmHg in microbead + PBS group (*p*** **<** **.01) and 22.17** **±** **3.19** **mmHg in microbead group (*p*** **<** **.01). There were no significant statistical differences between microbead + PBS group and microbead group. These results confirmed that polystyrene microbeads injection could increase IOP successfully and miRNA/NP-BRZ could reduce the IOP sustainably (Figure 5).

### Efficiency of nanoparticles in optic nerve protection

miRNA-124 was found to be neuroprotective and could effectively reduce the neuro-destructive of RGCs in glaucoma. TUNEL staining was adopted to detect the number of RGCs. TUNEL assay was used to compare and contrast the same area in the three groups to show the number and density of RGCs clearly. In order to evaluate the efficacy of miRNA/NP-BRZ in depth, we applied qRT-PCR method to detect *Thy1.1*, an important marker of RGCs, before and after drug treatment as well as during the administration period. ONC is another method of simulating the mechanical damage of RGCs and can lead to a significant reduction in the survival rate of the ganglion cell layer (GCL) cells.

### Improvement of survival rate of Tuj-1^+^ cells

**Figure 6.**
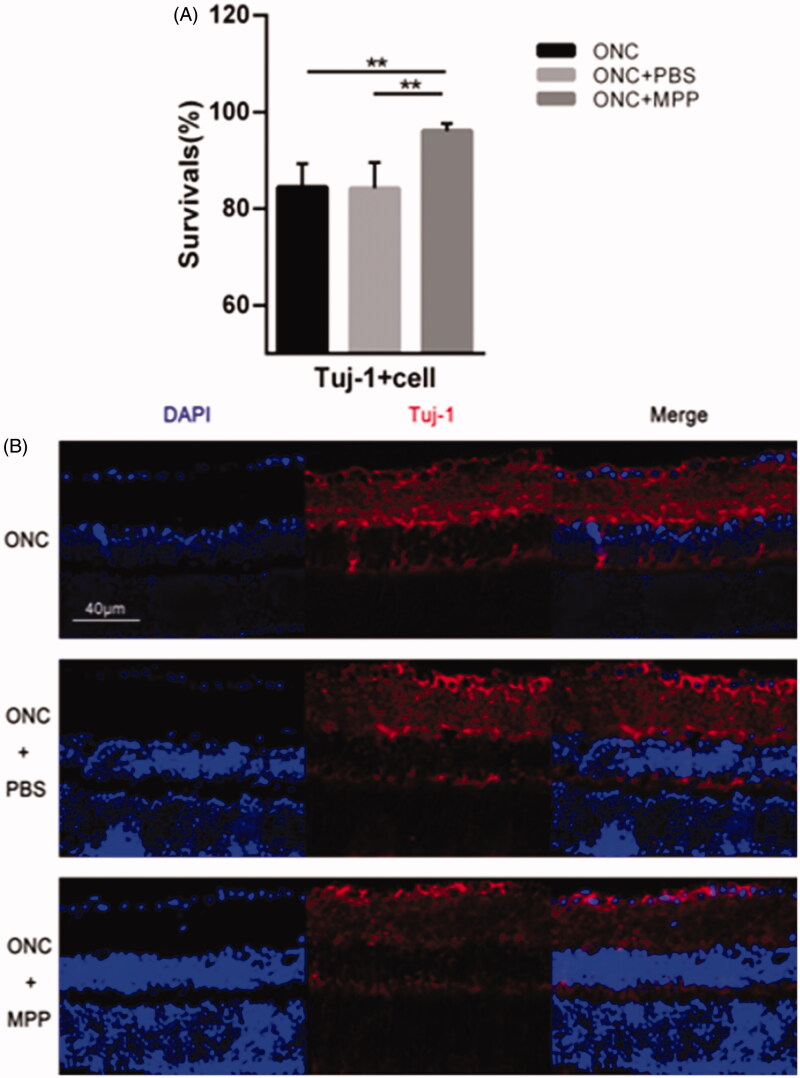
(A) Comparison of the survival rate between ONC and ONC + PBS and ONC + MPP; (B) The results of Tuj-1 immunofluorescence staining. After the miRNA/NP-BRZ treatment, the survival rate of Tuj-1^+^ cells in ONC + miRNA/NP-BRZ group was much higher than that in ONC group (**p** **<** **.01) and ONC + PBS group (**p** **<** **.01). There was no significant difference between ONC group and ONC + PBS group (*p*** **>** **.05). Scale bar: 40** **μm. Statistical significance was set at *p*** **<** **.05. miRNA/NP-BRZ: drug-loaded nanoparticles; ONC: optic nerve crush; PBS: phosphate-buffered saline.

Progressive RGC loss occurs in the ONC model as reported previously (Li et al., 1999; Leon et al., 2000; Sarikcioglu et al., 2007; Leung et al., 2008). β-III-tubulin (Tuj-1), expressed in cytoplasm and axons of active RGCs, is a typical marker used to detect RGCs. On day 3 after crush, the apparent reduction in the survival rate of Tuj-1^+^ cells could be observed after the ONC surgeries in each group (5 animals per group). As shown in Figure 6(A,B), compared to the free drug, ONC + miRNA/NP-BRZ (MPP) group (96.09** **±** **1.48%) showed better survival rate. There is no remarkable difference (*p*** **>** **.05) between ONC group (84.40** **±** **4.93%, *p*** **<** **.01) and ONC + PBS group (84.19** **±** **5.37%, *p*** **<** **.01). Therefore, it was concluded that miRNA/NP-BRZ can effectively improve survival rate of Tuj-1^+^ cells GCL.

**Figure 7.**
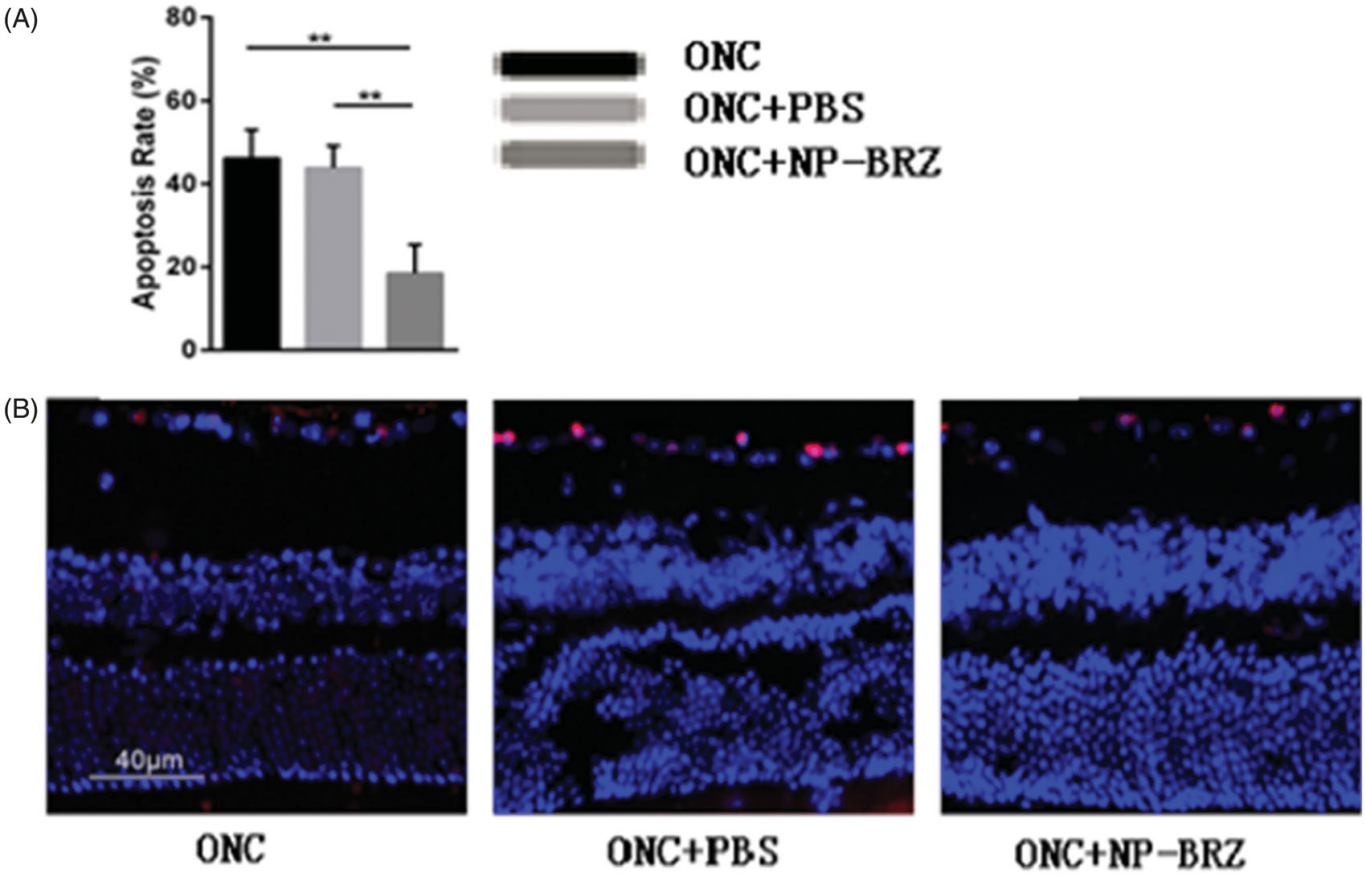
(A) Comparison of apoptosis rate in three groups; (B) fluorescent staining of retinal sections on day 3 following ONC using TUNEL assay. Only the light red stained cells in the GCL of retina were counted as TUNEL positive cells and the cellular nucleus were stained by light blue DAPI. The apoptotic rate of cells in the RGC layer was remarkably reduced in miRNA/NP-BRZ group compared to that of ONC group (**p** **<** **.01) and ONC + PBS group (**p** **<** **.01), which was significantly reduced by miRNA/NP-BRZ treatment.

On day 3 after surgery, the TUNEL assay showed that the apoptotic rate of RGCs in GCL was 18.50** **±** **6.94% in the ONC + miRNA/NP-BRZ group, which was significantly lesser than that in the ONC + PBS group (43.86** **±** **5.31%, *p*** **<** **.01) and ONC group (46.12** **±** **6.89%, *p*** **<** **.01), as shown in Figure 7(A,B). These data indicated that the co-delivery of miRNA/NP-BRZ remarkably inhibited RGCs apoptosis compared to the control and free drug groups. In addition, TUNEL assay was used to perform morphological observation and cell counting.

### Characteristics of RGCs

**Figure 8.**
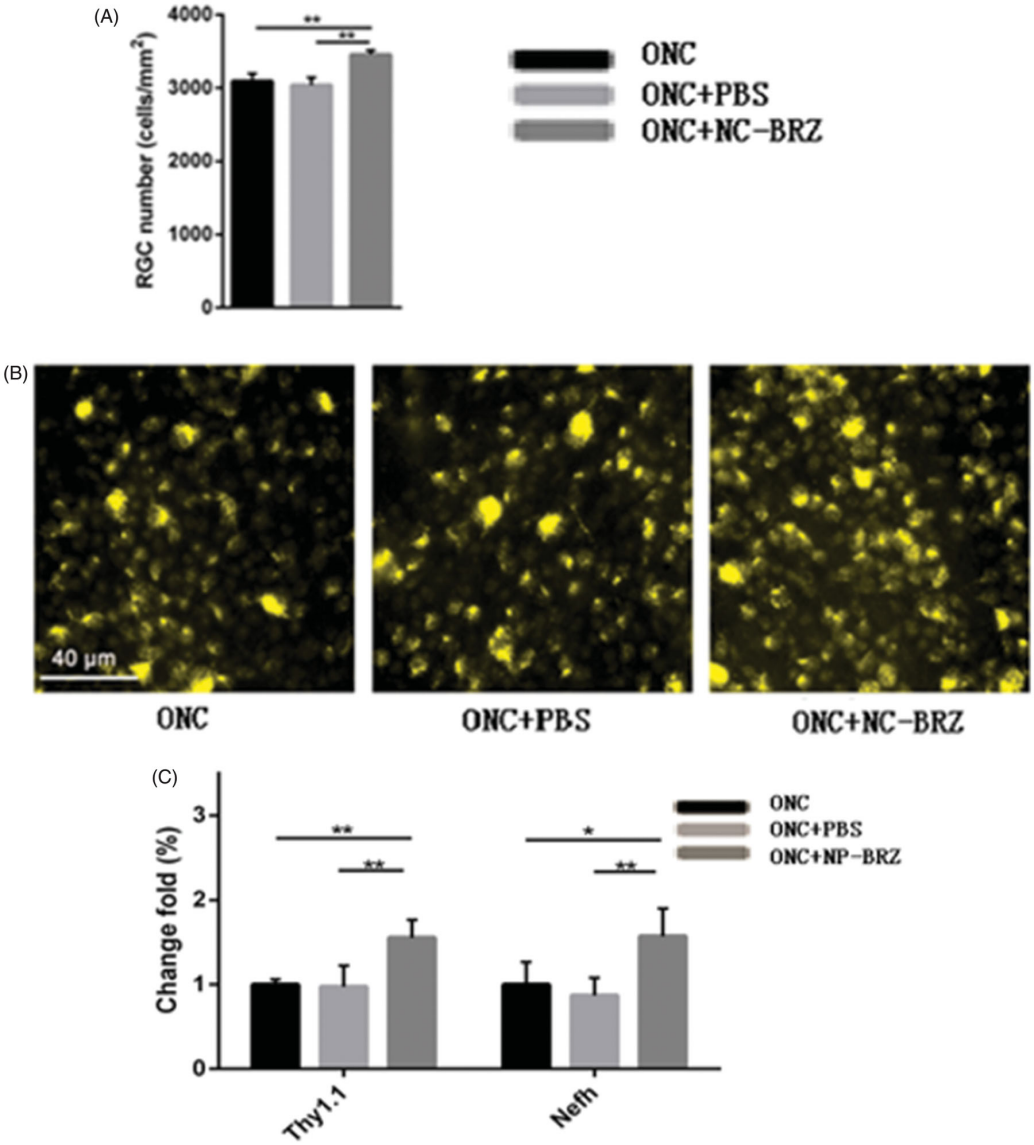
(A) Comparison of RGC numbers in three groups; (B) the results of the RGC density using the fluorogold labeling. The density of surviving RGCs in ONC + miRNA/NP-BRZ group was much higher than that in ONC group (**p** **<** **.01) and ONC + PBS group (**p** **<** **.01). There was no significant difference between ONC group and ONC + PBS group (*p*** **>** **.05). (C) The expression levels of RGC-selective genes were upregulated after miRNA/NP-BRZ injection. The retina mRNA expressions of Thy1.1 and Nefh were all significantly increased in ONC + miRNA/NP-BRZ group on day 3 after ONC when compared to ONC group and ONC + PBS group (**p** **<** **.01, *p** **<** **.05). miRNA/NP-BRZ treatment improved the mRNA expressions of Thy1.1 and Nefh in ONC + miRNA/NP-BRZ group when compared to ONC group and ONC + PBS group. Scale bar: 40** **μm. Statistical significance was set at *p*** **<** **.05. miRNA/NP-BRZ: drug-loaded nanoparticles; ONC: optic nerve crush; PBS: phosphate-buffered saline.

The assessment of the RGC density using the fluorogold labeling method showed similar results (Figure 8(A,B)). In the ONC + miRNA/NP-BRZ group, the density of fluorogold labeled RGCs was 3454.86** **±** **60.98 cells/mm^2^. The density of the surviving RGCs was significantly higher in the ONC + miRNA/NP-BRZ group than in the ONC group (3454.86** **±** **60.98 versus 3093.34** **±** **104.80 cells/mm^2^, *p*** **<** **.01); and in the ONC + PBS group (3454.86** **±** **54.27 versus 3037.81** **±** **104.27 cells/mm^2^, *p*** **<** **.01), whereas the mice in ONC group did not show a significant difference from those in ONC + PBS group. These results demonstrate that miRNA/NP-BRZ has effective cellular protective effect on RGCs.

To further confirm the observed partial protection of RGCs by miRNA/NP-BRZ treatment after ONC-induced damage, we also evaluated the expression levels of RGC-selective genes, i.e. *Thy1* and *Nefh*, on day 3 after ONC damage by qRT-PCR approach. The change in the expression levels of *Thy1* and *Nefh* is associated with various diseases, in particular, neurodegenerative disorders. Therefore, the detection of marker genes is an important strategy in the early diagnosis of diseases as well as the discovery of new drug targets. As shown in Figure 8(C), the expression of miRNAs *Thy1.1* and *Nefh* was all significantly upregulated in retina by 1.55** **±** **0.20, 1.57** **±** **0.33-fold on day 3 after miRNA/NP-BRZ injection compared to the ONC group, respectively (*n*** **=** **15, *p*** **<** **.01). The expression of *Thy1.1* and *Nefh* was not significantly upregulated after PBS injection compared to the ONC group, respectively (*n*** **=** **15, *p*** **>** **.05). These results confirmed that RGCs were partially rescued from ONC-induced cell loss or damage by a single intravitreal injection of miRNA/NP-BRZ.

### miRNA/NP-BRZ toxicity

**Figure 9.**
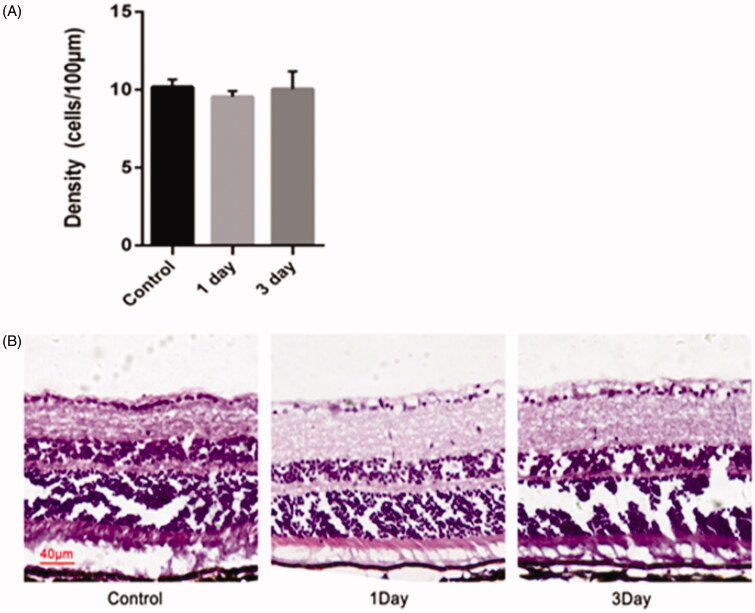
Morphological changes and cell density of RGCs in the retina of mice after an intravitreal injection of miRNA/NP-BRZ. There was no significant difference in the retina on day 1 and day 3 after miRNA/NP-BRZ injection compared to the normal retina. Abnormal changes in cell morphology was not observed in the 3 groups. Scale bar: 40** **μm; Statistical significance was set at *p*** **<** **.05; miRNA/NP-BRZ, drug-loaded nanoparticles.

Another objective of the co-delivery of miRNA/NP-BRZ is to diminish the toxicity to the eye tissue. There was no obvious toxic effect on retina in the control group as seen in Figure 9. The experimental group showed representative samples of H&E staining from the miRNA/NP-BRZ treated groups on day 1 and day 3. And, we observed that the morphological structure of RGCs in the retina was normal compared to the control group. The number of H&E stained cells in the GCL was 9.55** **±** **0.37 cells/100** **µm on day 1, 10.05** **±** **1.13 cells/100** **µm on day 3 which are close to that of the control group (10.18** **±** **0.48 cells/100** **µm). The statistical results showed that there was no significant difference in the number of RGCs in the 3 groups. Therefore, our co-delivery system could remarkably reduce the adverse effects of delivering medicine directly to the fundus of the eye via vitreous injection.

## Discussion

A major challenge in ocular therapeutics is to overcome the physiological barriers that separate the eye from the rest of the body (Kim et al., 2006). The poor bioavailability of drugs for ocular disorders demand optimization of drug delivery systems that can cross barriers (Kim et al., 2006, 2009). The major obstacles for the drug treatments are the presence of a restrictive blood–retina barrier (BRB), which limits the drug entry into ocular tissues and results in undesired irreversible neuronal damage. Recent advances in nanotechnology have revealed that novel nano-platforms are greatly promising in enhancing drug delivery while being intrusive less to the eye systems (Leon et al., 2000; De Jong & Borm, 2008; Kataki et al., 2015). The nanoscale drug-loading system in ophthalmic medication enables effective drug delivery as it can penetrate the BRB and hence, overcome the aforementioned challenges in the ocular disorder therapeutics (Kataki et al., 2015). It also improves the bioavailability of drugs significantly, reduces the dosage as well as usage frequency of drugs, and enhances the safety and longevity of the drug (De Jong & Borm, 2008). Hence, miRNA/NP-BRZ system offers a safer approach for treating retinopathy without direct contact with the retina.

Brinzolamide (BRZ), an effective aqueous humor inhibitor, can reduce IOP by suppressing the activity of key enzymes, carbonic anhydrase II (CA-II), in the formation of aqueous humor (Iester, 2008). Based on the intraocular tracing experiment, the BRZ in the nanoparticles designed by us could deliver BRZ accurately to target sites and can maintain lasting efficiency. The administration of BRZ nanoparticles results in anti-hypertensive effect compared to control group and stabilizes the IOP to a certain range permanently. MiRNA-124 as the most abundantly distributed in neurons play a critical role in the CNS in bringing about neuroprotective effects as well as promoting neuronal differentiation (Makeyev et al., 2007; Mishima et al., 2007; Sun et al., 2015). It is evident that the level of miRNA-124 is decreased in the cerebrospinal fluid of neurodegenerative patients compared to the controls. In addition, its expression was also found to be down-regulated in the cortex of neurodegenerative patients (Sun et al., 2015). As reported, miRNA-124 can target signal transducer and activator of transcription 3 (STAT3) and decrease IL-6 production and down-regulates the production of TNF-α converting enzyme (TACE) followed by reduction of TNF-alpha release (Sun et al., 2013). Makeyev et al. reveal that miRNA-124 promotes neurite outgrowth during neuronal differentiation (Makeyev et al., 2007). These imply the importance of its existence in the protecting nervous system. In our research, miRNA-124 and BRZ in the NPs were administered intravitreally to drive neuronal protection and reduce the IOP. To ensure that the new formula of nanoparticles is effective in lowering IOP and has a neuroprotective effect on RGCs, we constructed glaucoma models by using ONC as well as high IOP models induced by polymer microbeads injection. Pharmacokinetic evaluation tests, pharmacodynamics verifications of the experiments, and toxicity test further confirmed the feasibility of application of nanoparticle drug-loading system in the treatment of glaucoma. The neuroprotective effect of miRNA/NP-BRZ was shown in the following aspects: 1) the retina treated with miRNA/NP-BRZ had no obvious difference from normal retinas on the morphological structure; 2) the number of active RGCs marked by Tuj-1 antibody increased significantly; 3) the RGCs in groups treated with miRNA/NP-BRZ were averagely distributed; and 4) the neuroprotective effect was confirmed at the genetic level (*Thy1.1* and *Nefh* genes) via performing qRT-PCR. The antigen Thy1.1 exists on the surfaces of several kinds of cells and its presence is considered to be a sign of mature RGCs. Nefh, which encodes for neurofilament heavy polypeptide, is also a biomarker of neuronal injury. The significant increase in the expression of gene markers Thy1.1 and Nefh indicates that they could have been associated with neuronal maturation. Therefore, this co-delivery system clearly displays the neuronal protection and with functional improvement on the optic nerve damage.

In conclusion, we found that miRNA/NP-BRZ system was safe, nontoxic, and significantly efficient in reducing IOP and neuroprotection. Besides, the novel nanoparticles used in our study have the desired properties of nanoparticles including sustained-release effect. We expect the effect of the intravitreal administration of miRNA/NP-BRZ to be a potent therapeutic strategy to reduce the risk of vision loss from glaucoma treatment. However, the predicted functional roles of this co-delivery system in this study are merely a starting point for the study of miRNA-124 in glaucoma, and the exact mechanism and clinical uses of combination between the miRNA-124 and brinzolamide identified require further evidence and experimental verification.

## Data Availability

Data available within the article or its supplementary materials.
